# The Family Physician, the Specialist and the Patient

**Published:** 1903-09-19

**Authors:** 


### The Family Physician, the Specialist and the Patient.

Dr. Louis Weigel, of Rochester, New York, has recently taken as the theme of a presidential address at the American Orthopædic Association the important relations which exist between the family physician, the specialist and the patient as they occur at present in the United States. The family physician, or as we should call him the general practitioner, in America seems almost destined to disappear, and his extinction might well cause alarm to his brethren in Great Britain. Nor should the alarm be confined to the threatened class, for the uses of the general practitioner are great and manifold. At the present time there is a tendency in many families to employ three or four doctors where their fathers and mothers had only a single medical attendant in whom they placed implicit trust. Nowadays there is a doctor for the mother, another for the children, a third for the adults, and perhaps the father has another for himself. And if this is true in America there is very little doubt that it is equally true in England at the present time. The change is disastrous, but patients and doctors alike are to blame. The doctors, speaking from the surgical side at any rate, often fail to make a sufficiently detailed examination of their patients. They are content rather with symptoms and with such indications as are afforded by the pulse and the thermometer than with the actual physical signs of disease. This is perhaps more common in abdominal cases, in joints, and in malignant disease, and it leads to bad results by preventing prophylaxis or the early application of curative methods. All too often a perforated appendix is left until diffuse peritonitis has occurred because the pain is easier and the temperature has not risen; an injured joint is kept at rest until adhesions are formed; a dislocation is overlooked because no systematic examination of the joint has been made; or a lump in the breast is left until the axillary glands are extensively involved because the doctor agreed with the patient that there was no harm in it as it was not painful. And yet the general practitioner is not to blame wholly, for he is aided and abetted by the patient. The belief in the "old doctor," so inimitably expressed by Lucas Galen in his "Hospital Sketches," is by no means confined to the lower classes. A Grumbler, loquitur: "No, *'e* ain't like the old doctor as used to sit 'ere. *'E* used to sit with 'is chair up agin the wall and 'is feet on the table, and 'e 'ardly asked yer any questions, but 'e always did yer good. This doctor strips yer half naked, and examines yer all over, and then 'e don't look as if 'e knew what's the matter wid yer." But with the passing of the general practitioner there is no counterbalancing gain in the multiplication of the specialists. Whilst he is young the specialist lacks the general knowledge which gave to the old doctor his power of healing; when he is old and has gained experience his mind's eye is so focussed upon the particular organ to which his life's work has been devoted that he is apt to see it to the exclusion of everything else. The specialist is good therefore in operation and for consultation, but he often proves a most unsafe guide to those who put their faith in him on every occasion when they happen to be ill and who seek his advice when they should confide themselves to some practitioner of much wider outlook. It is obvious that a warning of this kind is needed, for there is a widespread belief that certain specialists of the baser sort have so little conscience that patients sent to them are lost for ever to those who send them, and it is obvious therefore that such persons do not always limit themselves strictly to the practice of their own speciality.

Yet it is difficult to suggest a remedy; Dr. Weigel pleads that the specialist should begin as a general practitioner, since a period of experience in a hospital can never be considered equivalent to the probation of general practice. In this country, however, the different classes of the profession are kept so widely distinct that only in a few country towns can a man thus emerge from one class into another, but when he does so he is often singularly successful. Some of our great hospitals solve the difficulty in another manner, for they make their assistant physicians and assistant surgeons take charge of, and teach in, the various special departments. Such men obtain a first-rate training, for they are subjected to the constant questioning of their students, they are visited by experts from all parts, their special department is only a part of their general medical or surgical work, and as they are picked men from the beginning there is but little fear that their ideas will become stereotyped or that their field of vision will become limited. It is in this manner that surgeons must look to become specialists rather than by a probationary period passed in general practice.

